# Spider Knottin Pharmacology at Voltage-Gated Sodium Channels and Their Potential to Modulate Pain Pathways

**DOI:** 10.3390/toxins11110626

**Published:** 2019-10-29

**Authors:** Yashad Dongol, Fernanda C. Cardoso, Richard J. Lewis

**Affiliations:** Division of Chemistry and Structural Biology/Centre for Pain Research, Institute for Molecular Bioscience, The University of Queensland, Brisbane 4072, Australia; y.dongol@imb.uq.edu.au (Y.D.); f.caldascardoso@imb.uq.edu.au (F.C.C.)

**Keywords:** chronic pain, ICK peptide, knottins, Na_V_, spider venom, voltage-gated sodium channel

## Abstract

Voltage-gated sodium channels (Na_V_s) are a key determinant of neuronal signalling. Neurotoxins from diverse taxa that selectively activate or inhibit Na_V_ channels have helped unravel the role of Na_V_ channels in diseases, including chronic pain. Spider venoms contain the most diverse array of inhibitor cystine knot (ICK) toxins (knottins). This review provides an overview on how spider knottins modulate Na_V_ channels and describes the structural features and molecular determinants that influence their affinity and subtype selectivity. Genetic and functional evidence support a major involvement of Na_V_ subtypes in various chronic pain conditions. The exquisite inhibitory properties of spider knottins over key Na_V_ subtypes make them the best venom peptide leads for the development of novel analgesics to treat chronic pain.

## 1. Introduction

Spiders are considered the most speciose and successful terrestrial venomous predators [1]. They comprise 119 families, 4141 genera and 48,255 species at the time of writing [2], with over 150,000 species estimated to exist [3,4]. They form the seventh most diverse order Araneae and completely rely on predation [3,5]. Their venoms comprise highly evolved venom peptides that facilitate both predatory behaviour by killing or paralysing the prey and defence against predation [6]. Medically significant cases of spider envenomation are less common and usually associated with intrusion in the spider’s natural habitat or threatening encounters [7]. Spider venoms are highly specialized in targeting the molecular receptors, especially the neuronal system, of insects to immobilize or kill their preys. However, their venoms also impart noxious effects to higher organisms such as mammals. The conserved structure and function of targeted receptors from evolutionarily distant prey species, such as insects and threatening species, including mammals, likely explains the ability of spider venom peptides to potently modulate human receptors [8,9].

Recent advances in analytical technologies have made comprehensive biochemical and functional investigations of spider venoms feasible [10]. In addition, high-throughput technologies, such as fluorescence imaging and automated electrophysiology, have sped up the screening and discovery of novel bioactive venom peptides. Chemically, spider venoms comprise a highly complex cocktail of enzymatic and non-enzymatic protein and peptide toxins and low molecular weight organic compounds, such as nucleotides, free amino acids, biogenic amines, neurotransmitters, acylpolyamines, inorganic ions and salts [11,12,13]. Spider venom peptides modulate an array of ion channels and receptor proteins, including transient receptor potential (TRP) channels, acid sensing ion channels (ASICs), mechanosensitive ion channels (MSICs), ionotropic glutamate receptors (GluRs), G-protein coupled receptors (GPCRs), voltage-gated sodium (Na_V_) channels, voltage-gated potassium (K_V_) channels, voltage-gated calcium (Ca_V_) channels and calcium-activated potassium channels (KCa) [4,14]. Interestingly, approximately one-third of the then described spider venom ion channel modulators targeted Na_V_ channels [15].

Venom peptides from spiders have been important tools in defining the function and pharmacology of Na_V_ channels and in elucidating binding sites in these channels [16,17,18]. For example, the hNa_V_ activator spider knottin Hm1a from *Heteroscodra maculata* recently elucidated the role of Na_V_1.1 in mechanical hypersensitivity and chronic visceral pain [19,20], while other Na_V_ inhibitor spider knottins continue to be developed as novel analgesics [21]. Thus, spider venoms provide a rich source of bioactive peptides to probe the function and pharmacology of Na_V_ channels as well as being leads to new therapeutics. In this review, we provide an overview of spider knottin pharmacology at Na_V_ channels, describe the structural determinants driving their potency and selectivity and discuss the potential of spider knottins to target Na_V_ subtypes involved in chronic pain conditions.

## 2. Voltage-Gated Sodium Channel Function and Structure

Voltage-gated sodium channels (Na_V_1.1–1.9) are transmembrane channel proteins selective to Na^+^ ions. They open upon depolarization of the membrane to allow the influx of Na^+^ ions and inactivate rapidly through a process named fast inactivation before returning to the closed state upon membrane hyperpolarization. Such rapid influx of Na^+^ ions is key to generation and propagation of action potential and underlies transmission of a wide array of somatosensory signals, including touch, smell, temperature, proprioception and pain [21]. A range of molecules discovered from natural sources (e.g., venomous animals) interact with Na_V_ channels to activate or inhibit the influx of Na^+^ ions [16,22]. Na_V_ channels are also expressed in non-excitable cells where they contribute to non-canonical functions [23], such as catecholamine release [24], angiogenesis [25], phagocytosis, endosomal acidification and podosome formation [26,27], production of pro-inflammatory mediators [28] and are key regulators in various human pathologies, such as cancer progression [29], multiple sclerosis [30], epilepsy [31] and pain syndromes [32,33]. In addition, mutations of Na_V_ channel-encoding genes contribute to diseases such as epilepsy, pain-related syndromes (e.g., inherited primary erythromelalgia (IEM), congenital insensitivity to pain (CIP) and paroxysmal extreme pain disorder (PEPD)) and cardiac arrhythmias, such as Brugada syndrome, atrial fibrillation and slow ventricular conduction [34,35,36,37,38].

Structurally, eukaryotic Na_V_ channels are complex transmembrane glycosylated proteins composed of a large pore-forming core protein (α-subunit, approximately 260 kDa) associated with one or more regulatory proteins (β-subunits, approximately 35 kDa) [39]. The α-subunit is primarily involved in Na^+^ conductance, whereas β-subunits modulate the Na^+^ current kinetics and α-subunit expression [40]. Four regulatory β-subunits (β1–β4) have been identified so far with a soluble splice variant β1B [41]. The β1 and β3 subunits make non-covalent interactions, while the β2 and β4 subunits make covalent interactions with the α-subunit to form a heteromeric protein [42].

**Figure 1:**
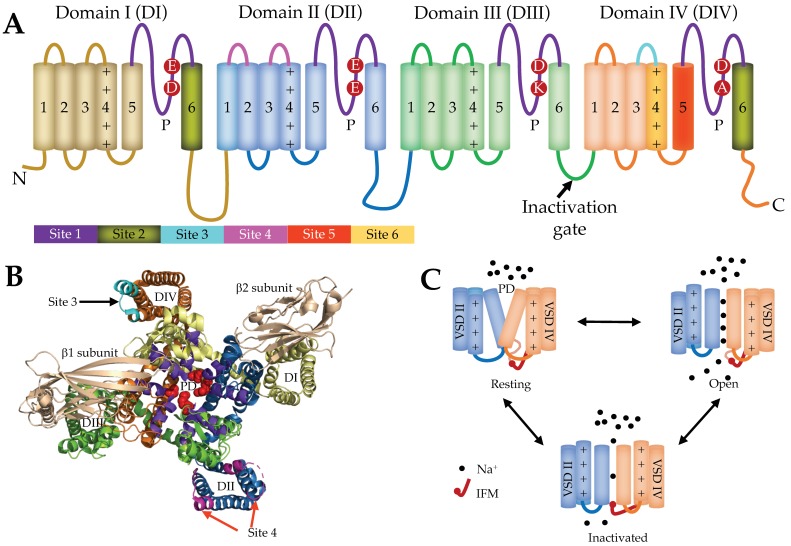
(**A**) Schematic representation of the α-subunit of voltage-gated sodium (Na_V_) channel. Four non-identical domains (DI–DIV) feature six neurotoxin receptor sites (Sites 1–6) and key residues contributing to the outer Na^+^ ion selectivity filter (EEDD) and inner selectivity filter (DEKA). The connecting S5–S6 linker is called P-loop (P) which together with S5 and S6 segments from each domain contributes in forming a Na^+^ ion selective channel pore. (**B**) Three-dimensional NMR structure of the Na_V_1.7 channel (PDB 6J8G) [55]. Four voltage sensing domains (VSDs), DI (yellow), DII (blue), DIII (green), and DIV (orange), are shown with their corresponding pore-forming segments (S5 and S6) arranged to form the pore domain (PD) selective to Na^+^ ions. The P-loop that contributes to forming the inner selectivity filter is coloured in red spheres (DEKA) and outer selectivity filter (EEDD) is coloured in purple. The S6 segments of all the four domains contribute to form the intracellular region of the pore. Site 3 (cyan) and Site 4 (pink) are the major binding sites for spider knottins. The β1 and β2 subunits which interact with DIII and DI, respectively, are highlighted in beige colour. (**C**) Schematic of the gating cycle of Na_V_ channels. At polarized potentials, the DI–DIV S4 segments are drawn towards the intracellular side due to the positive gating charges to render the closed conformation (down state). Upon depolarization, the forces holding the down state are relieved and DI–DIII S4 segments are rapidly released extracellularly to open the S6 channel gate in the open conformation (up state). The DIV S4 moves up slowly compared to DI–DIII S4 and drives the fast inactivation, where the channel is occluded intracellularly by the Ile, Phe, and Met (IFM) motif. After cell repolarization, the channel returns to a closed (resting) state [56,57,58].

The α-subunit comprises 24 transmembrane segments organized into four homologous, non-identical domains DI–DIV, each containing six transmembrane segments S1–S6 (Figure 1A,B) [43,44,45]. The S1–S4 segments of each domain contribute to the voltage sensing domain (VSD), and the S5 and S6 segments along with the extracellular connecting loop (P-loop) form the pore domain (PD) and selectivity filter. Conserved positively charged residues (arginine) at every third position in the S4 segment sense voltage changes across the membrane and regulate the gating kinetics of the Na_V_ channel [46] through a “sliding helix” or “helical screw” mechanism [47,48,49]. At resting membrane potentials, the S4 segments (voltage sensors) are drawn into the membrane where their positively charged residues form ion pairs with negatively charged adjacent residues from S1, S2 and/or S3 segments [50]. However, when the negative membrane potential becomes more positive during membrane depolarization, the S4 segments of DI–DIII move outward, resulting in a conformational change that opens the channel pore followed by the outward movement of DIV S4 that inactivates (blocks) the channel pore intracellularly [50,51]. Channel inactivation is the third cardinal feature of Na_V_ channels in addition to voltage sensing across the membrane and the selective Na^+^ filter [52]. Structurally, the cytoplasmic DIII–DIV linker forms a hinge that facilitates the inactivation and is key to the fast inactivation mechanism. A cluster of hydrophobic amino acids (i.e., Ile, Phe, and Met (IFM motif)) function as a hydrophobic latch to stabilize the inactivated state, and mutations in these residues individually and together alter the kinetics of fast inactivation [53]. Besides this motif, residues in the S4–S5 linkers of DIII (e.g., A1329) and DIV (e.g., N1662) are crucial for the fast inactivation as they form the docking site for IFM motif [54].

Although the Na_V_ channel gating has multiple kinetic states [59,60], it can be simplified into three distinct physiological states, the resting (closed), open, and inactivated, which develop from the voltage-sensitive conformational changes that occur within the α-subunit (Figure 1C) [57,61]. Toxins and drugs that interact with Na_V_ channels often bind preferentially to one of these conformational states to alter Na^+^ conductance or the gating properties of the channel. Six neurotoxin receptor sites (Sites 1–6) have been identified on the Na_V_ channel. Site 1 neurotoxin physically occludes the channel pore, whereas neurotoxins acting at Sites 2–6 affect the gating mechanisms of the channel. Venom peptides target four neurotoxin receptor sites in human Na_V_ channels, namely, Site 1 (e.g., µ-conotoxins), Site 3 (e.g., scorpion α-toxins, sea anemone toxins and spider toxins), Site 4 (e.g., scorpion β-toxins, spider toxins) and Site 6 (e.g., δ-conotoxins) [16,62]. Given their physico-chemical properties, it is not surprising that Na_V_ modulatory venom peptides preferentially target the extracellular side of VSDII (Site 4) and VSDIV (Site 3) [14,63].

## 3. Spider-Venom ICK Peptides

Inhibitor cystine knot (ICK) peptides have a disulphide-rich structural motif that forms a “knot” that confers high structural, thermal and proteolytic stability, making them attractive starting points for structure–function studies and clinical lead development [64]. This structural motif comprises at least three disulphide bonds with connections between C1–C4, C2–C5 and C3–C6, where two disulphide bonds form a ring threaded by the third (C3–C6) disulphide bond to form the knot. These scaffolds were first described as “knottins” in 1980s [65] and later identified as a “cystine knot” in the crystal structure of nerve growth factor [66,67]. Pallaghy et al. [68], in 1994, coined the term “inhibitor cystine knot” to identify the cystine knot motif with a triple-stranded anti-parallel β-sheet topology. Craik et al. [69] further categorized these disulphide-rich structural motifs as (i) growth factor cystine knots (GFCKs), (ii) inhibitor cystine knots (ICKs) and (iii) cyclic cystine knots (CCKs). ICKs and CCKs have the same disulphide connectivity, but the disulphide connectivity in GFCK differs where C1–C2 threads the ring formed by C2–C4 and C3–C6. ICKs and CCKs are referred to as knottins and cyclotides, respectively [70]. Animal toxin cystine knots have an ICK structural motif [69].

**Figure 2:**
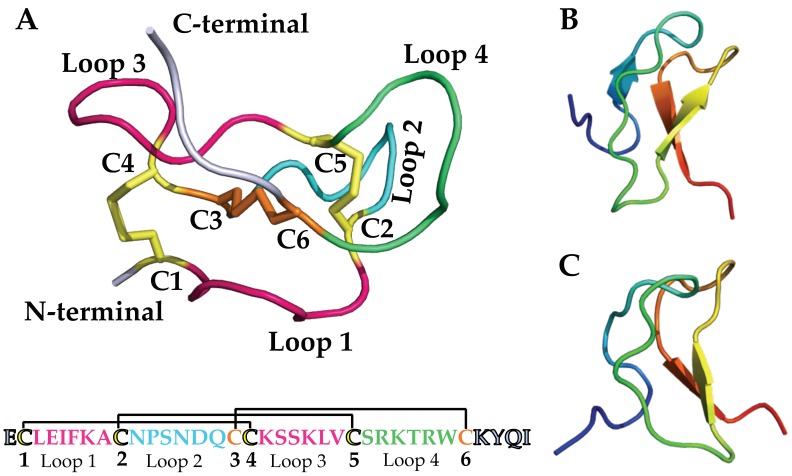
(**A**) **Top:** Spider venom knottin HwTx-IV (PDB: 2m50) [83] demonstrating the cystine knot motif with three disulphide bridges. A ring structure made up of two disulphide bridges, C1–C4 and C2–C5 (yellow), and the intervening peptide backbone (pink) penetrated by a third disulphide bridge, C3–C6 (orange), to form a pseudo-knot. The three-disulphide bonds form four loops (pink, green and cyan). **Below:** The primary structure of HwTx-IV with three disulphide bridges and four loops. (**B**,**C**) Spider venom knottins with varying β-sheet topology. The colour from the N-terminal to the C-terminal follows the rainbow spectrum from blue to red. (**B**) HnTx-IV (PDB: 1NIY) [85] comprises three β-sheets, whereas (**C**) CcoTx-I (PDB: 6BR0) [86] comprises two β-sheets.

**Figure 3:**
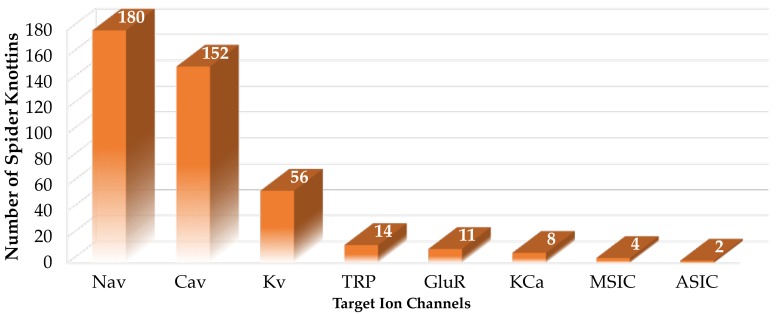
Number of spider venom knottins modulating ion channels. The data were collected from the UniProt database on 23 September 2019 using the following search descriptors: “voltage gated sodium channel impairing spider ICK toxin” for Na_V_ channel targeting spider knottins, “voltage gated calcium channel impairing spider ICK toxin” for voltage-gated calcium (Ca_V_) channel targeting spider knottins, “voltage gated potassium channel impairing spider ICK toxin” for voltage-gated potassium (K_V_) channel targeting spider knottins, “TRP impairing spider ICK toxin” for transient receptor potential (TRP) channel targeting spider knottins, “ionotropic glutamate receptor impairing spider ICK toxin” for ionotropic glutamate receptor (GluR) targeting spider knottins, “calcium-activated potassium channel impairing spider ICK toxin” for calcium-activated potassium (KCa) channel targeting spider knottins, “mechanosensitive ion channel impairing spider ICK toxin” for mechanosensitive ion channel (MSIC) targeting spider knottins and “ASIC impairing spider ICK toxin” for acid-sensing ion channel (ASIC) targeting spider knottins [84].

Spider venoms are a rich source of disulphide-rich peptides, including knottins [4]. With few exceptions, such as the atracotoxins, they display the ICK features comprising three disulphide bonds (Figure 2A). However, certain variations are observed within the β-sheet topology (Figure 2B,C). Unlike the triple-stranded anti-parallel β-sheet topology defined in knottins, spider venom knottins typically comprise two β-strands with a few exceptions displaying a third strand at the N-terminal [71]. Besides the disulphide bridge connectivity, another conserved structural feature of spider venom knottins is the hydrophobic patch surrounded by charged amino acids on the toxin’s surface [72,73] that contributes to potency and selectivity [74,75,76,77,78,79,80,81,82,83]. The UniProt database lists 747 entries on search term “spider venom ICK toxin” (23 September 2019) that are further categorized into their ion channel targets (Figure 3) which shows approximately 43% of the thus far described ion channel-impairing spider knottins targeted Na_V_ channel [84].

The first discovery of Na_V_-modulating spider venom peptides was in the mid-1980s, when Fontana and Vital-Brazil [87] demonstrated that the crude venom of *Phoneutria nigriventer* is capable of activating Na_V_ channels in muscle and nerve cells. Later in 1991, Rezende et al. [88] isolated three neurotoxic fractions (PhTx1, PhTx2 and PhTx3) from *P. nigriventer* venom, and the most toxic fraction (PhTx2) was later shown by Araújo et al. [89] in 1992 to inhibit the Na_V_ channel inactivation. In 1989, Adams et al. [90] isolated μ-agatoxins (I–VI) from *Agelenopsis aperta* which became the first spider venom with disulphide-rich peptides (8 cysteines arranged into 4 disulphide bridges) targeting Na_V_ channels to induce repetitive firing in the neurons. Since then, a journey of three decades of research on spider venom peptides modulating Na_V_ channels resulted in the discovery of a number of venom peptides which have been applied into research on the mechanisms of Na_V_ channel modulation and potential therapeutics. Based upon the level of sequence identity and inter-cysteine spacing, these Na_V_ channel-targeting spider toxins were classified into 12 families (NaSpTx1–12) [15].

### 3.1. Pharmacology of NaSpTx

The spider knottins’ interaction with Sites 3 and 4 displayed diverse pharmacological phenotypes (Table 1 and Table 2). Broadly, they either (i) prevent channel opening in response to membrane depolarization by trapping the VSD II in the closed state; (ii) facilitate channel opening by trapping VSD II in open state or (iii) prevent channel inactivation by binding DIV S4 in the closed state to impair the movement of the inactivation gate [15,18,63]. Curiously, an integrated pharmacology of Site 3 and Site 4 indicates multiple binding site interactions by the same toxin [91,92]. For example, the toxic fraction PhTx2 from the venom of *P. nigriventer* not only prolonged the inactivation and deactivation (Site 3 phenotype) of the Na_V_ channel but also shifted the voltage dependence of activation (Site 4 phenotype) and steady-state inactivation towards negative potentials (Site 3 phenotype) [89]. Later, PnTx2–6 alone confirmed this complex pharmacology was achieved by a single toxin [93]. Further evidence of dual pharmacological profile includes versutoxin (VTX) from *Hadronyche versuta*, which besides the classical Site 3 features, such as delaying the channel inactivation and shifting the voltage dependence of inactivation towards more negative potential, also displayed the classical Site 4 feature of reducing the maximum (peak) sodium current [94].

Detailed characterization of the dual effects of the spider knottin JzTx-XI from *Chilobrachys jingzhao* on hNa_V_1.5 revealed a concentration dependence in the modulation of Na_V_ channels. At low concentrations (≤90 nM), JzTx-XI significantly reduced the peak currents (inhibition of channel activation; Site 4) but at higher concentrations (≥180 nM), besides reducing the peak currents, it also slowed the current decay (fast inactivation; Site 3) [91]. Further, these modulatory effects were demonstrated by Df1a from *Davus fasciatus* over hNa_V_ subtypes 1.1–1.7, where the toxin shifted the voltage-dependence of activation and steady-state fast inactivation of the hNa_V_ subtypes to more hyperpolarizing potentials, with exception of depolarizing shifts in activation of hNa_V_1.3 and hNa_V_1.7 and depolarizing shifts in the inactivation of hNa_V_1.3 [92]. Furthermore, the toxin delayed the fast inactivation along with the reduction of peak currents in hNa_V_1.1, hNa_V_1.3 and hNa_V_1.5 [92]. Such subtype-varying profiles have also recently been reported for Pn3a (*Pamphobeteus nigricolor*) [61], Pre1a (*Psalmopoeus reduncus)* [111] and JzTx-14 (*C. jingzhao*) [125]. These multi-site effects of spider knottins on Na_V_ channels need to be carefully considered when establishing their pharmacological profiles.

Another channel state modulated by spider knottins is channel inactivation [91,93,94,98,116]. For example, when tested over tetrodotoxin sensitive (TTX-S) Na_V_ channels on rat dorsal root ganglia (DRG) neurons, HnTx-III and HnTx-IV from *Selenocosmia hainana* differed in their channel repriming kinetics. More specifically, HnTx-III delayed channel recovery from inactivation, whereas HnTx-IV had no effects [116]. Similarly, steady-state inactivation can also be differently modulated by spider knottins. For example, JzTx-35 (*C. jingzhao*), which selectively targeted hNa_V_1.5 similarly to JzTx-III (*C. jingzhao*), differed by shifting the steady-state inactivation of the channel towards more hyperpolarized potentials [112]. The hyperpolarizing shift in the voltage dependence of steady-state inactivation stabilizes the channel in the inactivated state [128]. However, potent inhibitor toxins like HwTx-IV (*Ornithoctonus huwena)*, ProTx-III (*Thrixopelma pruriens*) and HnTx-IV showed no effect on steady-state inactivation [80,114,129]. Likewise, the excitatory JzTx-I (*C. jingzhao*) delayed the channel inactivation of other Site 3-acting spider knottins including δ-ACTXs, but the peak current amplitude, I–V relationship and steady-state inactivation remained unaltered [101]. Such characteristics among the spider knottins provide novel paths to modulate Na_V_ channels.

Spider knottins also inhibit Na_V_ channel activation. ProTx-I and ProTx-II from *T. pruriens* showed, for the first time, the depression of Na_V_ channel activation by shifting the voltage dependence of activation towards more depolarized potentials [103]. This is in contrast to typical scorpion β-toxins which, although reduced the peak current, shifted the voltage dependence of activation and inactivation to hyperpolarized potentials [130]. Its structural homology with hanatoxin isolated from *Grammostola spatulata* suggested interactions with the DII S3–S4 linker to inhibit the channel activation [103,131]. Later, residues critical for these interactions were identified in the domain II of the hNa_V_ channel [132]. Spider knottins include a growing number of depressant toxins, including JzTx-V (*C. jingzhao*), Df1a (*D. fasciatus*), Pre1a (*P. reduncus*), PnTx4(5-5) from *P. nigriventer*, CcoTx-1 (*Ceratogyrus cornuatus*), PaurTx (*Phrixotrichus auratus)* and Cd1a (*Ceratogyrus darlingi*) which induce a depolarizing shift in voltage-dependence of activation at specific Na_V_ subtypes [73,79,92,107,110,111]. However, a number of spider depressant knottins reduced the sodium currents without changing the activation or inactivation kinetics (e.g., HwTx-IV, HnTx-III, HnTx-IV) [114,116,129], by shifting activation and inactivation to hyperpolarizing potentials (e.g., Df1a) [92] or by inhibiting both activation and inactivation (e.g., JzTx-14) [125]. Spider knottins (e.g., JzTx-V) also altered the slope factor of activation and inactivation associated to shifts in their voltage dependence [79,91] suggesting cooperativity between the four S4 segments or multiple binding sites [9,91].

Spider knottins display distinct affinities and modes of action for insect and mammalian Na_V_ channels. Magi 5 from *Macrothele gigas* interacted with Site 3 on the insect Na_V_ channels and Site 4 on mammalian Na_V_ channels to induce Na^+^ influx [133]. These observations support the concept of common binding “hot spots” proposed by Winterfield and Swartz (2000) [134], where binding sites in the voltage-gated ion channels are not independent structural sites. Recently, PnTx4(5-5) also showed a distinct affinity and mode of action on insect and mammalian Na_V_ channels [126]. On BgNa_V_ from the cockroach *Blatella germanica*, PnTx4(5-5) strongly slowed channel inactivation (EC_50_ 213 nM) and increased current amplitude, while it inhibited sodium currents of the mammalian Na_V_1.2–1.6 channels, with higher potency on Na_V_1.3. This unique selectivity and species-dependent mode of action provides new insight into the molecular mechanisms of spider gating modifier toxins [126].

Cell background can also influence pharmacological profiles of spider knottins [116,117,129]. For example, hyperpolarizing shifts (10−11 mV) in voltage-dependence of steady-state inactivation imparted by HnTx-III and HnTx-IV in TTX-S Na^+^ currents in rat DRG were not observed on heterologously expressed hNa_V_1.7. Similarly, HnTX-III which delayed the recovery from inactivation of TTX-S Na^+^ channels on rat DRG did not affect the repriming kinetics of heterologously expressed hNa_V_1.7 [116,117]. Indeed, the difference in the relative proportion of Na_V_ subtypes in different DRG cell types (small and large diameter) [117], species and age [117,135], β-auxiliary subunit combinations [136] and difference in the membrane lipid composition [82] likely influence the biophysical and pharmacological properties of Na_V_ subtypes. The pharmacological and biophysical properties of Na_V_ channels are also influenced by the expression system and species differences. For example, Df1a was approximately 8.5 fold less potent at hNa_V_1.7 expressed in *Xenopus* oocytes than at hNa_V_1.7 expressed in HEK 293 cells [92]. Similarly, Pn3a was approximately 2 fold less potent at rNa_V_1.7 and 5 fold less potent at mNa_V_1.7 compared to hNa_V_1.7 [61].

Interaction with membrane lipid is another important feature of spider knottins [14] that can influence affinity and potency [75,82,137]. The anionic charges in the polar head groups of the lipid surrounding Na_V_s probably increase the affinity of positively charged spider knottins, while hydrophobic residues in these toxins can make favourable electrostatic and hydrophobic interactions with the hydrophobic core of the lipid bilayer [138]. Although spider knottins like ProTx-I and ProTx-II exploit electrostatic interactions to increase their potency, HwTx-IV and Hd1a (*Haplopelma doriae*) rely less on membrane binding for potent inhibition [139,140]. Interestingly, a HwTx-IV analogue engineered to increase the affinity for lipid membrane showed improved inhibitory potency at hNa_V_1.7 [137]. On the other hand, the most potent and selective analogues of GpTx-1 (*Grammostola porteri*) and ProTx-II had reduced affinity for lipid bilayer [75]. Thus, a direct correlation between the toxin–lipid interaction and the potency or the selectivity for Na_V_s could not be established [86] beyond facilitating the positioning of the toxin at the membrane surface proximal to exposed Na_V_ residues [75]. Indeed, the conserved amphipathicity of the spider knottins may be an evolutionary adaptation favouring toxin promiscuity [86].

### 3.2. Structure–Function of NaSpTx

Structure–function studies highlight the role of knottin residues in determining the toxin potency and selectivity [14]. For example, the distribution of Ser4 and Asp5 at N-terminus instead of cationic Lys4 and Arg5 of δ-ACTX-Hv1b (*H. versuta*) shifted selectivity for mammalian Na_V_ channels [97]. Similarly, CcoTx-2 (D32Y-CcoTx-1) potently inhibited Na_V_1.3 (IC_50_ 88 nM), while CcoTx-1 was inactive at this subtype. Surprisingly, Asp32 or Tyr32 located on the side opposite the hydrophobic patch also influenced potency, suggesting that residues beyond the hydrophobic patch can also influence toxin–channel interactions [73]. In addition, the ICK fold retained by key mutants suggested that specific amino acid interactions between the toxin and channel are prime in Na_V_ channel modulation and subtype selectivity [83,85].

**Figure 4:**
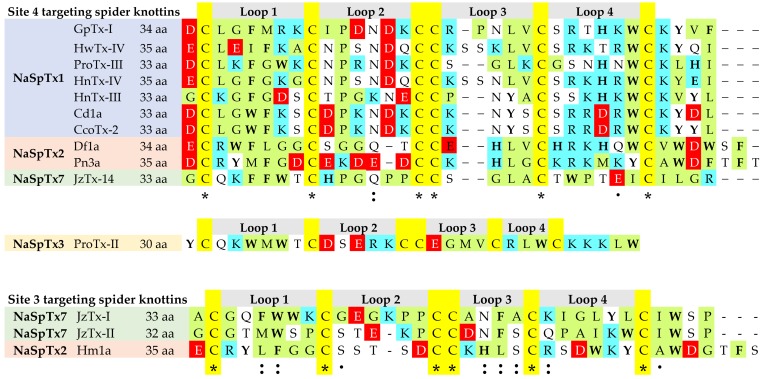
Multiple sequence alignment of spider knottins targeting Site 4 and Site 3 in Na_V_ channels. The NaSpTx family 1–3 generally target Site 4 to inhibit channel activity, except Hm1a which targets Site 3 to excite the channel [20]. JzTx-14 [125] from NaSpTx7 targets Site 4 and inhibits the channel, whereas JzTx-I [100] and JzTx-II [98] from NaSpTx7 targets Site 3 to excite the channel. Yellow highlights conserved cysteines, green highlights hydrophobic residues, cyan indicates positively charged residues, red indicates negatively charged residues and bold letter indicates the aromatic residues. The “*” indicates identical residues, “:” indicates strong conservation, “.” indicates weak conservation.

The NaSpTx family 1–3 incorporate most of the Na_V_-modulating spider knottins with promising therapeutic lead potential, including ProTx-II [103,141], ProTx-I [132], ProTx-III [80], Df1a [92], Pn3a [61], HwTx-IV [83,137,142,143], GpTx-I [76] and CcoTx-I [144]. Sequence alignments of selected Na_V_-modulating spider knottins targeting Sites 3 and 4 in Na_V_ channels are shown in Figure 4. Besides the shorter N-terminus and longer C-terminus in Site 3 and Site 4 targeting spider knottins, this alignment highlights conserved hydrophobic residues in loop 1 and positively charged residues in loop 4 of Site 4 targeting spider knottins. In contrast, negatively charged residues are distributed in loop 2 of NaSpTx1–3 spider knottins and in the N-terminal of NaSpTx families 1 and 2. The conserved Arg and Lys in loop 4 of NaSpTx1–3 appear to be crucial for inhibitory function of depressant spider toxins belonging to these families [145].

The C-terminal WCK motif is also conserved across well-characterized sodium channel-blocking toxins from NaSpTx families 1 and 3, suggesting that these residues are key determinants of activity [83]. In the cryogenic electron microscopy (cryo-EM) structure of ProTx-II–hNav1.7 VSDII-Na_V_Ab complex, the corresponding Trp24 served as a hydrophobic anchor to stabilize ProTx-II interactions with hNa_V_1.7 [58]. More specifically, the hydrophobic patch Trp5, Trp7, Trp24 and Trp30 stabilized binding by allowing deeper penetration into the membrane lipid [58]. In addition, the ProTx-II–hNa_V_1.7 cryo-EM structure confirmed a key role of the C-terminal basic residues capped by hydrophobic residues in anchoring the toxin into the membrane [58]. In contrast, JzTx-14 belonging to NaSpTx7 has a loop 4 that lacks positively charged residues and Arg serving as a C-terminal cap, but still inhibits eight out of nine Na_V_ channel subtypes at nanomolar concentrations [125]. It has additional hydrophobic residues in each of the four loops with only one acidic residue that suggest an alternative binding mode. Indeed, structure–function studies have shown that hydrophobic and aromatic residues in loop 1, loop 4 and C-terminus and positively charged residues distributed in loop 4 and C-terminus were critical for toxins’ affinity [83,85,117]. Interestingly, the highly Na_V_1.7-selective Pn3a, like other NaSpTx2 spider knottins, lacks positively charged residues in the C-terminus and instead contains hydrophobic and negatively charged Asp residues.

The overall net charge of the peptide also affects the toxin activity. For example, decreased net anionic charge in E1G, E4G, Y33W-HwTx-IV enhanced inhibition by 45 fold (IC_50_ 0.4 nM) compared to native HwTx-IV (IC_50_ 17 nM) [142], which was mostly driven by enhanced hydrophobic interactions associated to the Y33W mutation (IC_50_ 1.4 nM). Amidation of the C-terminus also has a direct influence on the potency of HwTx-IV [142]. While characterizing ProTx-III and Df1a interactions with Na_V_ channels, Cardoso et al. [80,92] elucidated the significance of C-terminal amidation in enhancing potency and altering subtype selectivity of spider toxins.

**Figure 5:**
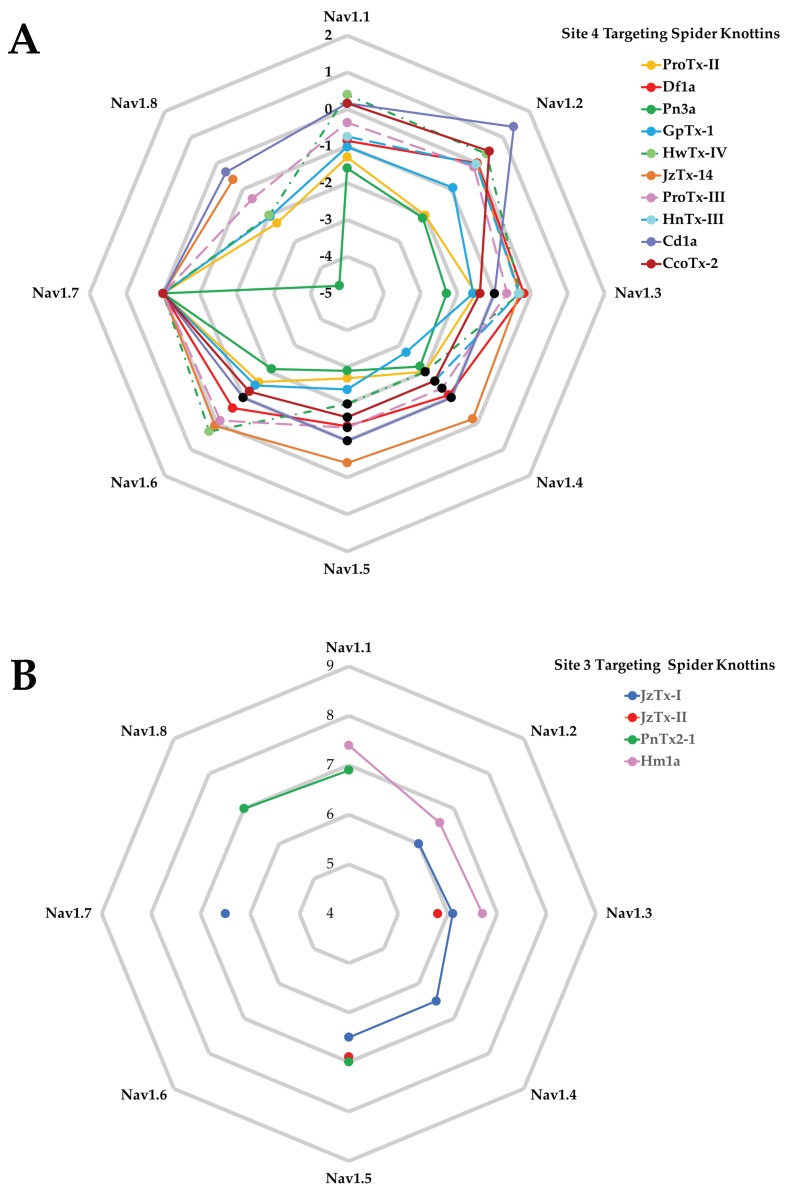
“Spider-plot” of Site 4 and Site 3 targeting spider knottins. (**A**) The pIC_50_ of Site 4 spider knottins normalized against Na_V_1.7 are shown. These data are from assays performed on human Na_V_ subtypes, except JzTx-14 which was performed at mammalian Na_V_ subtypes. Data for GpTx-1 [122], ProTx-III [80], Cd1a [110], and CcoTx-2 [110] were obtained from Fluorescence Imaging Plate Reader (FLIPR) experiments, while the remainder were acquired using electrophysiology. Black dots indicate that the IC_50_ values for the corresponding knottins were less potent than the value indicated. (**B**) The pEC_50_ of Site 3 targeting spider knottins are shown. JzTx-I [100] was tested on rat Na_V_1.2–1.4 and human Na_V_1.5 and Na_V_1.7. JzTx-II [98] was tested on rat Na_V_1.3 and human Na_V_1.5. PnTx2-1 [147] was tested on rat Na_V_1.1 and Na_V_1.8, and human Na_V_1.5. Hm1a [20] was tested on human Na_V_ subtypes.

The comparison of the potency of spider knottins over Na_V_1.1–Na_V_1.8 for Site 4 (Figure 5A) and Site 3 (Figure 5B) toxins highlights the limited pharmacological data for Site 3 spider knottins that are available mostly for Na_V_1.3 and Na_V_1.5. In contrast, Site 4 targeting spider knottins preferentially target hNa_V_1.7 with Cd1a and CcoTx-2 having clear preference over hNa_V_1.2, making this class an excellent starting point for the design of analgesic spider knottins. Indeed, the hNa_V_1.7 selective spider knottin Pn3a confirms this potential [61,146] and shows analgesic effects in acute postsurgical pain [146]. A more complete list of spider knottins showing potency across different Na_V_ channel subtypes and DRG are listed in Appendix A Table A1 and Table A2.

**Figure 6:**
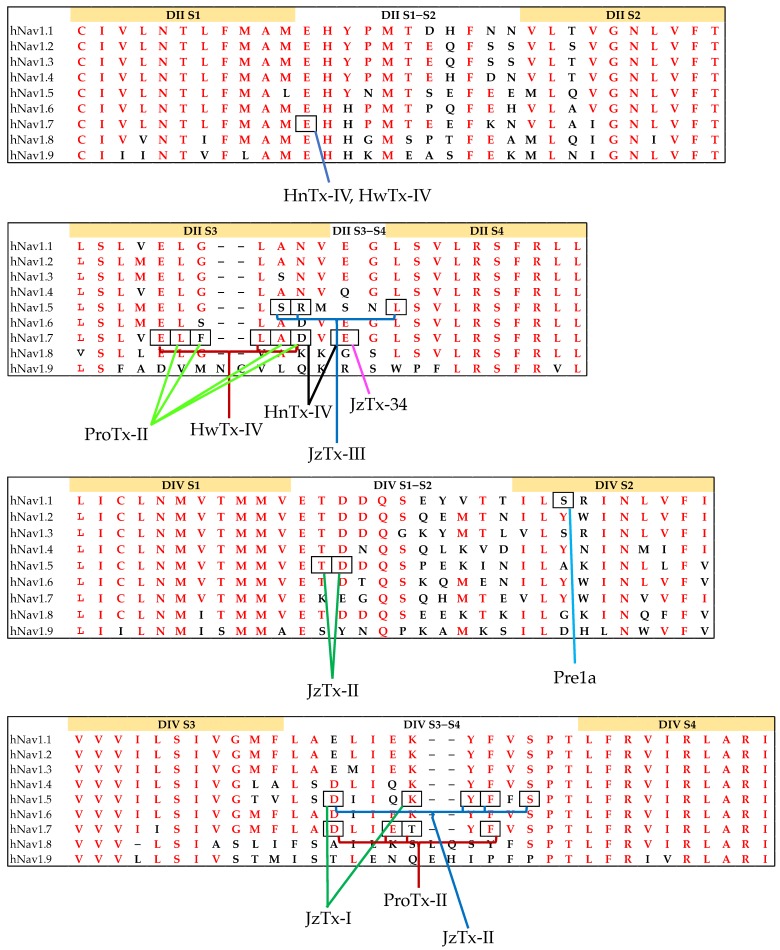
Sequence alignment of (**A**) DII S1–S2, (**B**) DII S3–S4, (**C**) DIV S1–S2 and (**D**) DIV S3–S4 of hNa_V_1.1–1.9. Identical residues among hNa_V_ subtypes are highlighted in red. hNa_V_1.9 demonstrates the highest variation compared to other hNa_V_ subtypes. Boxes highlights key residues in the interactions for the spider knottins HwTx-IV [149], HnTx-IV [129], ProTx-II [58,104], JzTx-III [106], JzTx-II [98], JzTx-I [100], JzTx-34 [119] and Pre1a [111].

Mutagenesis and chimera studies of Na_V_ channels are typically used to determine critical residues for toxin binding on hNa_V_ channels [92,129,148]. These studies have revealed why the spider knottins HnTx-III, HnTx-IV, and HwTx-IV show Site 1-like channel inhibition (pore blocker) although they are gating modifiers [113,116,128]. Specifically, the Y326S mutation in Na_V_1.7 decreased the channel sensitivity to tetrodotoxin (TTX) but not to HwTx-IV [114], while three residues (i.e., Glu753, Asp816 and especially Glu818) outside the pore were shown to be involved in the interactions of HnTx-IV with hNa_V_1.7 (Figure 6A,B) [129]. hNa_V_1.7/rK_V_2.1 S3–S4 paddle chimera studies revealed that Df1a primarily interacted with the DII voltage sensor of hNa_V_1.7 and had weaker interactions with VSDs of DIII and DIV [92]. Wingerd et al. [111] showed that the spider knottin Pre1a interacted with the DII and DIV S3–S4 loops of Na_V_1.7 as well as the S1–S2 loop of DIV with the latter interaction likely conferring subtype selectivity. They also showed the role of the serine residue in the DIV S2 helix of hNa_V_1.1 (Figure 6C) and rNa_V_1.3 in inhibiting the fast inactivation process in these channel subtypes [111]. Spider knottin Hm1a also targets DIV S3b–S4 and S1–S2 loops that likely underlie its subtype selectivity for hNa_V_1.1 [20]. 

Certain spider knottins can interact with multiple sites in the channel as demonstrated by ProTx-II’s interactions at DII and DIV of hNa_V_1.7 (Figure 6B,D) [55,104]. Furthermore, the DII residues, Glu753, Glu811, Leu814, Asp816 and Glu818, in hNa_V_1.7 that are critical for inhibition of activation by HwTx-IV are partially conserved in hNa_V_1.7 DIV. However, the partial conservation of DII residues in DIV (EgLDi) in wild-type hNa_V_1.7 made HwTx-IV interact specifically with DII [149]. Mutational studies further identified that Asp1609 in hNa_V_1.5 was crucial in determining the JzTx-II potency. However, rNa_V_1.8 and rNa_V_1.9 resistant to JzTx-II have Ala and Arg, respectively, instead of Asp1609 [98]. Similarly, Glu818 in hNa_V_1.7 is conserved in HnTx-IV-sensitive Na_V_ channels rNa_V_1.2 and rNa_V_1.3 but is replaced by a neutral amino acid in HnTx-IV-resistant rNa_V_1.4 and hNa_V_1.5 [129]. In addition, Schmalhofer et al. [132] showed the crucial role of Phe813 in ProTx-II’s selectivity for hNa_V_1.7 over other Na_V_ subtypes. Finally, cysteine palmitoylation, which is a common reversible lipid modification process vital for Na_V_ channel biosynthesis, is also associated with the affinity of spider knottins to Na_V_ channels [150]. It regulates the gating and pharmacology of WT-rNa_V_1.2a as observed by the hyperpolarizing shift in steady-state inactivation and slowing in the channel recovery from fast inactivation upon depalmitoylation of intracellular cysteines on both WT-rNa_V_1.2a and G1097C-rNa_V_1.2a [150].

## 4. Knottins for Na_V_s in Pain Pathways

Pain allows direct perception of noxious stimuli to avoid actual or potential tissue damage. Primary sensory neuron (nociceptor) signals are transmitted to the brain through action potentials generated by ion channels and receptors. Genetic and molecular studies in animals and humans identified six Na_V_ channel subtypes (i.e., Na_V_1.1, Na_V_1.3, Na_V_1.6, Na_V_1.7, Na_V_1.8 and Na_V_1.9) critical for the generation and transmission of pain-related signals [20,21,36,151,152,153,154,155,156,157,158]. The Na_V_1.7, Na_V_1.8 and Na_V_1.9 are preferentially expressed in the peripheral nervous system (PNS), while Na_V_1.1 and Na_V_1.6 are found in both the central nervous system (CNS) and PNS. The subtype Na_V_1.3 is generally expressed in the CNS and absent in the adult PNS but is re-expressed in peripheral pain-signalling pathways upon neuronal injury [21,154,159]. The PNS localization of Na_V_1.3, Na_V_1.7, Na_V_1.8 and Na_V_1.9 avoids reaching the CNS and inducing associated off-target side effects. Besides these four major peripheral targets, there is evidence of the involvement of Na_V_1.6 [160,161,162] and Na_V_1.1 [20] in various peripheral pain pathways, which suggests these might also be drug targets in chronic pain types.

### 4.1. Na_V_1.1

Votage-gated sodium channel subtype 1.1 (Na_V_1.1) is a TTX-S sodium channel encoded by the *SCN1A* gene located on human chromosome 2q24.3 [163]. They initiate action potential and repetitive firing in neurons and are expressed in both the CNS and PNS and including the colonic myenteric plexus [17,164]. In the PNS, Na_V_1.1 is predominantly expressed in medium-to-large diameter DRG neurons (A-fibres) but less expressed in small diameter unmyelinated neurons (i.e., C-fibres [165,166]) and has a smaller contribution in C-fibre-mediated nociceptive transmission [166,167]. However, in colonic afferents which predominantly comprise C-fibres, nearly 50% of the neurons are expressed Na_V_1.1 [168]. The hNa_V_1.1-selective activator Hm1a revealed its role in mechanical but not thermal hypersensitivity in the absence of neurogenic inflammation [20]. Recently, Salvatierra et al. [19] demonstrated the upregulation of Na_V_1.1 in chronic visceral hypersensitivity (CVH) and its inhibition reducing the mechanical pain in an irritable bowel syndrome (IBS) rodent model. Furthermore, Na_V_1.1 contributed to peripheral nerve injury-associated mechanical hypersensitivity [19].

Besides the role in nociception [19,20], Na_V_1.1 participates in the familial hemiplegic migraine type 3 [169]. In addition, the anti-epileptic drug rufinamide, a Na_V_1.1 inhibitor [170,171], alleviated spared nerve injury-evoked mechanical allodynia which also stabilizes Na_V_1.7 in the inactivated state [172]. In addition to the hNa_V_1.1-selective activator Hm1a, which identified a key role for this subtype in mechanosensitive pain, the spider knottins CcoTx-1, CcoTx-2, Df1a, ProTx-III, HwTx-IV and Pre1a inhibit Na_V_1.1 in the nanomolar range and are thus potential leads to novel analgesics (Table A1).

### 4.2. Na_V_1.3

Voltage-gated sodium channel subtype 1.3 (Na_V_1.3) is a TTX-S sodium channel encoded by the *SCN3A* gene located on human chromosome 2q24.3 [163]. It produces fast activating and inactivating sodium currents and ramp currents due to the fact of its slow closed-state inactivation. These contribute to neuronal hyperexcitability by reducing thresholds and enhancing the repetitive and ectopic firing in injured neurons [17,154,173]. This channel is primarily expressed in embryonic DRG neurons and absent in adult DRG, but re-expressed during peripheral nerve injury and painful neuromas [166,174,175]. It is also expressed in enterochromaffin cells in the large and small intestine of humans and mice, where they participate in responses to chemical and mechanical stimuli [164,176,177].

Neuropathic pain models such as sciatic nerve transection [178], spinal nerve ligation (SNL) [179], SNI [180] and chronic constriction injury (CCI) confirmed the upregulation of Na_V_1.3 [181]. Interestingly, this channel is upregulated only when peripheral projections are transected. The upregulation of Na_V_1.3 is also associated to hyperexcitability of small DRG neurons [178]. Furthermore, infraorbital nerve-chronic constriction injury (ION-CCI) produced significant upregulation of Na_V_1.3 and downregulation of Na_V_1.7, Na_V_1.8 and Na_V_1.9 in trigeminal nerves [182].

Intrathecal administration of Na_V_1.3-targeting antisense oligonucleotides attenuated Na_V_1.3 upregulation and consequently reduced hyper-responsiveness of dorsal horn neurons and mitigated pain behaviours following CCI [181]. Chen et al. [156] also observed increased expression of Na_V_1.3 in a CCI rat model in which neuropathic pain was alleviated by the intrathecal administration of MiR-96, a microRNA that inhibits Na_V_1.3 expression. Another microRNA miR-30b attenuated SNL-evoked neuropathic pain by targeting *SCN3A* and downregulating the expression of Na_V_1.3 mRNA and protein both in DRG neurons and the spinal cord [157].

The role of Na_V_1.3 is also demonstrated in inflammatory pain [183], where its expression was upregulated in DRG neurons of rats with diabetic neuropathy and showing mechanical allodynia and thermal hyperalgesia [184]. In this same model, Na_V_1.3 knockdown by adeno-associated virus (AAV)-shRNA-Na_V_1.3 vector reduced neuropathic pain [185]. *Varicella zoster* virus (VZV) infection causing post-herpetic neuralgia (PHN) also showed upregulation of Na_V_1.3 [186]. However, although Na_V_1.3 was downregulated by antisense oligonucleotides in an SNI model, mechanical and cold allodynia were not attenuated [180]. Similarly, the mechanical allodynia remained unaltered following nerve injury in nociceptor specific and global Na_V_1.3 knockout mouse models [187]. The druggability of Na_V_1.3 in chronic pain management requires Na_V_1.3-selective inhibitors [17]. Spider knottins ProTx-II, ProTx-III, HwTx-IV, HnTx-III, HnTx-IV and CcoTx-2 have nanomolar potency towards Na_V_1.3 and rational engineering may generate Na_V_1.3-selective leads (Table A1).

### 4.3. Na_V_1.6

Voltage-gated sodium channel subtype 1.6 (Na_V_1.6) is a TTX-S sodium channel encoded by the *SCN8A* gene which is located in human chromosome 12q13.13 [163]. They localize at the axon initial segment (AIS) and nodes of Ranvier in the CNS and PNS, including central projections and soma of the C-fibres [188,189,190]. It underlies persistent and resurgent sodium currents and repetitive neuronal excitability, with loss- or gain-of-function mutations reducing or increases the neuronal excitability, respectively [188]. Persson et al. [191] demonstrated the expression of Na_V_1.6 in the axons of small nerve bundles beneath the epidermis and in the nerve terminals of nociceptors. Its increased expression is observed in complex regional pain syndrome Type 1 (CRPS1), post-herpetic neuralgia (PHN) patients [162,192] and in diabetic neuropathy in mice [193]. Recently, gain-of-function mutation M136V in Na_V_1.6 was reported in trigeminal neuralgia with significantly increased peaks inward, resurgent currents and overall excitability of trigeminal nerves [194,195]. Deuis et al. [160] showed that oxaliplatin-induced cold allodynia is mediated by Na_V_1.6 expressed in peripheral pathways, suggesting a key role in cold pain pathways. Finally, the selective inhibitor Cn2-E15R developed from the Na_V_1.6 agonist Cn2 can be used to determine the extent of Na_V_1.6 contributions to pain behaviours [196].

Local knockdown of Na_V_1.6 alleviated spontaneous pain and mechanical allodynia imparted by the scorpion toxin BmK I [161]. Furthermore, local Na_V_1.6 knockdown reversed mechanical pain in an SNL rat model by reducing sympathetic sprouting around Na_V_1.6-positive neurons [197]. The Na_V_1.6 expression was also upregulated in a pain model of DRG inflammation where Na_V_1.6 knockdown reduced the pain behaviours and the abnormal bursting of the sensory neurons, including nociceptors [198]. Spider knottins modulating Na_V_1.6 at nanomolar concentrations include ProTx-II, HwTx-IV, JZTx-14, Pre1a and Df1a (Table A1).

### 4.4. Na_V_1.7

Voltage-gated sodium channel subtype 1.7 (Na_V_1.7) is encoded by the *SCN9A* gene which is located in human chromosome 2q24.3 [163]. It is preferentially expressed in large-and-small diameter DRG neurons, visceral sensory neurons, olfactory sensory neurons, trigeminal ganglia and sympathetic neurons [151,152,199]. In DRG neurons, they are present from the peripheral to central terminals, with higher expression in small-diameter DRG neurons (C-fibres) [200]. This channel produces a rapidly activating and inactivating TTX-S sodium current that slowly recovers from inactivation and limits the frequency of firing. The slower onset of closed-state inactivation also limits inactivation during sub-threshold depolarizations and facilitates robust action potential generation that can amplify sub-threshold inputs [152,153,201,202]. Besides action potential generation and propagation, Na_V_1.7 also contributes to neurotransmitter release in peripheral and central projections of sensory neurons [151,153].

Exclusive localization in the PNS and compelling genetic and functional evidences have centre-staged Na_V_1.7 in pain research [153,158,203,204]. For example, mutations have been identified for various gain-of-function pain disorders, such as inherited erythromelalgia (IEM) [204], paroxysmal extreme pain disorder (PEPD) [203], small fibre neuropathy (SFN) [158] and painful diabetic peripheral neuropathy [205]. The gain-of-function mutations lead to hyperpolarizing shifts in activation, increased amplitude of ramp current, impaired inactivation, increased persistent currents and enhanced resurgent currents, all contributing to the hyperexcitability of DRG neurons [152]. Similarly, recessively inherited loss-of-function mutations are linked to congenital insensitivity to pain (CIP), with loss of olfaction being the only known side effect [36]. In addition, Na_V_1.7 was upregulated in rodent models of visceral hypersensitivity [164]. These observations have supported Na_V_1.7 as a promising therapeutic target for pain [153]. Considering the recent opportunities for spider knottins to unravel the mechanisms of Na_V_ modulation, the discovery and engineering of spider knottins selectively inhibiting Na_V_1.7 provided exciting new opportunities for the development of novel pain therapeutics [135]. A number of spider knottins (Table A1) show nanomolar potency to inhibit Na_V_1.7, with several undergoing optimization through saturation mutagenesis, directed evolution and/or rational engineering to enhance the potency and selectivity [14].

### 4.5. Na_V_1.8

Voltage-gated sodium channel subtype 1.8 (Na_V_1.8) is a TTX-R sodium channel encoded by the *SCN10A* gene in human chromosome 3p22.2 [163] and preferentially expressed in nociceptive DRG and trigeminal neurons (>90%) as well as in low-threshold mechanoreceptors [206,207], skin free nerve terminals [162], and corneal neurons [208]. It generates slowly inactivating rapidly repriming TTX-R sodium currents with a depolarized shift in the voltage dependence of activation and inactivation [154,209,210]. The channel also contributes to slow resurgent currents that probably underlie the excitability of nociceptors in DRG [211]. It also contributes significantly to the action potential upstroke and are sometimes referred to as overshoot channels [212].

The role of Na_V_1.8 in inflammatory pain has been well documented. It had increased expression in DRG neurons when carrageenan was injected into rat hind paw [213] and in cultured DRG neurons treated with inflammatory mediators [214,215]. Similarly, Beyak et al. [216] showed increased Na_V_1.8 currents in an animal model of colitis. Notably, inhibition of inflammatory pain induced by complete Freund’s adjuvant (CFA) was observed in antisense-mediated Na_V_1.8 knockdown in rats [217]. In addition, the upregulation of Na_V_1.8 in a mouse model of bowel obstruction underpins its role in visceral hypersensitivity [218]. However, its role in neuropathic pain is not well defined with studies demonstrating downregulation of Na_V_1.8 mRNA, protein and currents in the sciatic nerve after axonal transection [219,220,221]. On the other hand, increased Na_V_1.8 levels were reported in spared axons and neuronal cell bodies of uninjured nerves [222,223]. This discrepancy is probably associated to the effects of inflammatory mediators in neuropathic pain models [154]. Finally, gain-of-function mutations of Na_V_1.8 identified in painful neuropathy cases suggest its role in peripheral neuropathy [224]. Although a few spider toxins such as ProTx-I, ProTx-II and JxTx-14 targeted Na_V_1.8 at nanomolar concentrations, only the spider knottin Hl1a shows selectivity towards Na_V_1.8 (Table A1).

### 4.6. Na_V_1.9

Voltage-gated sodium channel subtype 1.9 (Na_V_1.9) is encoded by the *SCN11A* gene located in the human chromosome 3p22.2 [163]. These TTX-R channels are preferentially expressed in nociceptive DRG neurons, trigeminal ganglia and myenteric neurons [225,226], where they are activated only at hyperpolarized potentials near resting membrane potential to produce ultra-slow inactivating and persistent sodium currents [227]. These biophysical properties assist in amplifying subthreshold stimuli, lowering the threshold for single action potentials, and increasing repetitive firing [151,227,228].

The role of Na_V_1.9 has been demonstrated in inflammatory pain with increased Na_V_1.9 current density and lower thresholds for action potential generation that ultimately enhances neuronal excitability [151,214,229]. Indeed, Na_V_1.9 knockout mice showed diminished mechanical hypersensitivity to formalin and CFA and failed to develop thermal hyperalgesia upon CFA or carrageenan injection [151,230,231,232]. Rare dominant gain-of-function mutations in *SCN11A* have been reported in a number of human pain disorders, such as familial episodic pain [233], painful small fibre neuropathy [234,235] and insensitivity to pain [155]. Despite the challenges in obtaining heterologous expression of Na_V_1.9, chimeras Na_V_1.9/K_V_2.1 showed ProTx-I interacted with Na_V_1.9 S3b–S4 paddle motif and potentiated Na_V_1.9 currents in rat DRG [236].

## 5. Conclusions and Future Directions

In addition to a pivotal role of Na_V_1.7 in pain processing, other Na_V_ subtypes including Na_V_1.1, 1.3, 1.6, 1.8, and 1.9 are increasingly showing supportive and/or pivotal roles in various acute and chronic pain conditions. A large range of spider knottins modulate the Na_V_ function and continue to be developed as potential analgesic leads. In addition, spider knottins provide exquisite research tools to further explore the role of Na_V_ channels and how they can be modulated. Given that multiple sodium channels often contribute to chronic pain conditions, therapeutic leads targeting multiple Na_V_ channels could be advantageous; for example, chronic visceral pain may be best treated by a Na_V_1.1/Na_V_1.7/Na_V_1.8 inhibitor, while diabetic neuropathy may be best treated by a Na_V_1.3/Na_V_1.6/Na_V_1.7 inhibitor. However, despite Na_V_-inhibiting spider knottin potential to target multiple subtypes, most have been optimized for potency and selectivity towards a single channel subtype hNa_V_1.7 [14]. This review summarises spider knottin Na_V_ channel pharmacology that might be useful in guiding structure–function studies and the rational design of multi-valent spider knottin leads towards the development of therapeutic leads for chronic pain management.

## Figures and Tables

**Table 1:** Pharmacological features of Site 3 interacting spider knottins resulting in delay of channel inactivation.

Features	Examples
Hyperpolarizing shift in voltage-dependence of activation	PhTx-2 [89], VTX [94], Hv1 [95], Ar1 [96], Hv1b [97], PnTx2-6 [93], JzTx-II [98]
Hyperpolarizing shift in steady-state inactivation	VTX [94,99], PhTx-2 [89], Ar1 [89], Hv1b [97], PnTx2-6 [93]
No significant effect in voltage-dependence of steady-state inactivation	JzTx-I [100,101], JzTx-II [98]
Reduced peak inward current amplitude	VTX [94], Ar1 [96], Hv1b [97], PnTx2-6 [93]
No change in peak inward current amplitude	JzTx-I [100]
Increased peak inward current amplitude	Hm1a [20]
Increased recovery rate from inactivation	VTX [94], Ar1 [96], JzTx-I [102], JzTx-II [98]
Decreased recovery rate from inactivation	PnTx2-6 [93]

**Table 2:** Pharmacological features of Site 4 interacting spider knottins resulting in reduction of peak inward current.

Features	Examples
Depolarizing shift in voltage-dependence of activation	ProTx-I [103], ProTx-II [77,103,104], JzTx-III [105,106], CcoTx-I [73], CcoTx-2 [73], CcoTx-3 [73], PaurTx-3 [73], JzTx-V [79,107], JzTx-IX [108], Hm-3 [109], Cd1a [110], Pre1a [111], Pn3a [61], Df1a [92], JxTx-XI [91], JzTx-35 [112]
No effect in voltage-dependence of activation	HwTx-IV [104,113,114,115], HnTx-III [116,117], JzTx-34 [118,119], Hm-1 [120], Hm-2 [120], Hd1a [121], GpTx-1 [122], Hl1a [123], PnTx1 [124], ProTx-III [80], Pre1a [111], JzTx-14 [125]
Hyperpolarizing shift in voltage-dependence of steady-state inactivation	HnTx-III [116], HnTx-IV [116], JzTx-V [79], Hm-1 [120], Hm-2 [120], JzTx-35 [112], PnTx4 (5-5) [126], Df1a [92], JzTx-34 [119]
Delay in channel inactivation	ProTx-II [104], JzTx-XI [91], Df1a [92], JzTx-14 [125]
Decreased channel recovery from inactivation	HnTx-III [116], JzTx-XI [91], Pn3a [61]
No effect in channel recovery from inactivation	HnTx-IV [116], JzTx-34 [118], HnTx-III [127], Hd1a [121]
Hyperpolarizing shift in voltage-dependence of activation	Df1a [92]
Depolarizing shift in voltage-dependence of steady-state inactivation	Df1a [92]

## Appendix A

**Table A1:** Inhibitory effects of spider knottins on Na_V_ channel subtypes. Values presented were obtained from electrophysiological experiments unless otherwise as stated.

Toxin	NaSpTx Family	Na_V_1.1	Na_V_1.2	Na_V_1.3	Na_V_1.4	Na_V_1.5	Na_V_1.6	Na_V_1.7	Na_V_1.8	Na_V_1.9	Others
ProTx-I	2							51 nM (*n*, X, *h*); 72 nM (*s*, X, *h*); [103]	27 nM (*n*, X, *r*) [103]		
ProTx-II	3	15.8 nM (*s*, C, *h*) [141]	52.9 nM (*n*, H, *rNav1.2a*) [104] 41 nM (*n*, H, *h*) [132] 79.4 nM (*s*, H, *h*) [141]	109.9 nM (*n*, H, *r*) [104] 102 nM (*n*, C, *h*) [132] 25 nM (*s*, *h*) [141]	107.6 nM (*n*, H, *r*) [104] 39 nM (*n*, H, *h*) [132] 79.4 nM (*s*, H, *h*) [141]	29 nM (*s*, H, *h*) [103] 79.4 nM (*n*, H, *h*) [104] 79 nM (*n*, H, *h*) [132] 398 nM (*s*, H, *h*) [141]	26 nM (*n*, H, *h*) [132] 31.6 nM (*s*, C, *h*) [141]	0.7 nM (*n*, H, *h*) [104] 0.3 nM (*n*, H, *h*) [132] 0.8 nM (*s*, H, *h*) [141]	19 nM (*n*, X, *r*) [103] 146 nM (*n*, H, *h*) [132]		
GP-W7Q-W30L ProTx-II	3	6310 nM (*s*, C, *h*) [141]	794 nM (*s*, H, *h*) [141]		15,849 nM (*s*, H, *h*) [141]	>3162 nM (*s*, H, *h*) [141]	2512 nM (*s*, C, *h*) [141]	10 nM (*s*, H, *h*) [141]			
ProTx-III	1	60 nM, *rec-G*; 101 nM, *s-OH*; 11.3 nM, *s-NH*_2_ (H, *h*) [80]		21.9 nM, *rec-G*; 41.3 nM, *s-OH*; 11.5 nM, *s-NH*_2_ (H, *h*) [80]		>500 nM, *rec-G* and *s-OH*; >50 nM, *s-NH*_2_ (H, *h*) [80]		9.5 nM, *rec-G*; 11.5 nM, *s-OH*; 2.5 nM, *s-NH*_2_ (H, *h*) [80]			
HwTX-IV	1	41 nM (*n*, H, *h*) [237]	150 nM (n, H, *r*) [114] 10 nM, *s-NH2*, 54 nM, *rec* (H, *h*) [83] 44 nM (*n*, H, *h*) [237]	338 nM (*n*, H, *r*) [114] 190 nM (*n*, H, *h*) [237]	400 nM (*n*, H, *r*) [114] >10,000 nM (*n*, H, *h*) [237]	>10,000 nM (*n*, H, *h*) [114] >10,000 nM (*n*, H, *h*) [237]	52 nM (*n*, H, *h*) [237] 83.3 nM (*n*, H, *h*) [237]	22.7 nM (*n*, H, *h*) [104] 26 nM (*n*, H, *r*) [114] 16 nM, *s-NH2*, 55 nM, *rec* (H, *h*) [83] 17 nM (*s*, H, *h*) [142] 32.4 nM (*s*, S, *h*, FLIPR) [137] 100 nM (*n*, H, *h*) [237] 32.6 nM (*n*, H, *h*, Manual ephys) [237]	>10,000 nM (*n*, H, *h*) [237]		30 nM (*n*, TTX-S currents, rat DRG) [113]
E1G-E4G-Y33W HwTx-IV	1	8.4 nM (*rec*, H, *h*) [143] 1100 nM (*rec*, H, *h*, FLIPR) [143]	11.9 nM (*rec*, H, *h*) [143] 540 nM (*rec*, H, *h*, FLIPR) [143]	7.2 nM (*rec*, H, *h*) [143] 1400 nM (*rec*, H, *h*, FLIPR) [143]	369 nM (*rec*, H, *h*) [143] >30,000 nM (*rec*, H, *h*, FLIPR) [143]	Insensitive up to 1000 nM (*rec*, H, *h*) [143] >30,000 nM (*rec*, H, *h*, FLIPR) [143]	6.8 nM (*rec*, C, *h*) [143] 600 nM (*rec*, H, *h*, FLIPR) [143]	0.4 nM (*s*, H, *h*) [142] 3.3 nM (*rec*, C, *h*) [143] 5100 nM (*rec*, H, *h*, FLIPR) [143]	Insensitive up to 1000 nM (*rec*, C, *h*) [143] >30,000 nM (*rec*, H, *h*, FLIPR) [143]		
E1G-E4G-F6W-Y33W HwTx-IV	1							7.55 nM (*s*, S, *h*, FLIPR) [137]			
HnTx-I	1		68,000 nM (*n*, X, *r*) [145]					>10,000 nM (*s*, H, *h*) [238]			No inhibition of TTX-S and TTX-R currents up to 100,000 nM [145]
G7W-N24S HnTx-I	1							440 nM (*rec*, C, *h*) [81]			
E1G-N23S-D26H-L32W HnTx-I	1							3.6 nM (*s*, H, *h*) [238]			
HnTx-III	1	1270 nM (*n*, H, *h*) [117]	275 nM (*n*, H, *h*) [117]	491 nM (*n*, H, *h*) [117]	No activity [117]	No activity [117]		232 nM (*n*, H, *h*) [117]			1.1 nM (TTX-S rat DRG); No inhibition in TTX-R currents [116]
HnTx-IV	1		36.1 nM (*n*, H, *r*) [129]	375 nM (*n*, H, *r*) [129]	>10,000 nM (*n*, H, *r*) [129]	No inhibition up to 1000 nM [129]		21 nM (*s*, *n*, H, *h*) [127]			44.6 nM (TTX-S rat DRG) [116] 34 nm (TTX-S rat DRG) [85]
JzTx-III	7		No effect [106]	No effect [106]	No effect [106]	348 nM (*rec*, H, *h*) [106]	No effect [106]	No effect [106]			380 nM (*n*, rat cardiac myocytes) [105]
JzTx-V	3			292 nM (*s*, H, *h*) [79]	5.12 nM (*s*, H, *r*) [79]	2700 nM (*s*, H, *h*) [79]		61 nM (*s*, H, *h*) [79]			27.6 nM (*n*, TTX-R currents, rat DRG); 30.2 nM (*n*, TTX-S currents, rat DRG) [107]
JzTx-IX	2				5420 nM (*n*, H_T_) [108]	450 nM (*n*, H_T_) [108]					650 nM (*n*, TTX-R currents, rat DRG); 360 nM (*n*, TTX-S currents, rat DRG) [108]
JzTx-XI	2					124 nM (*n*, C) [91]					
JzTx-14	3		194 nM (*n*, H_T,_ *M*) [125]	426.3 nM (*n*, H_T,_ *M*) [125]	290.1 nM (*n*, H_T,_ *M*) [125]	478 nM (*n*, H_T,_ *M*) [125]	158.6 nM (*n*, H_T,_ *M*) [125]	188.9 nM (*n*, H_T,_ *M*) [125]	824 nM (*n*, H_T,_ *M*) [125]		
JzTx-34	2	No inhibition (*s*, H_T_, *r*) [119]	No inhibition (*s*, H_T_, *r*) [119]	7950 nM (*s*, H_T_, *r*) [119]	No inhibition (*s*, H_T_, *r*) [119]	No inhibition (*s*, H_T_, *r*) [119]	No inhibition (*s*, H_T_) [119]	610 nM (s, H_T_, *h*) [119]	No inhibition (*s*, ND cells, *h*) [119]		85 nM (*rec*, TTX-S currents, rat DRG); No inhibition in TTX-R currents [118] 91 nM (*s*, TTX-S currents, rat DRG); No inhibition in TTX-R currents [119]
JzTx-35	2					1070 nM (*n*, H, *h*) [112]					
CcoTx-1	1	523 nM (*n*, X) [73] 1060 nM (*n*, H, *h*, FLIPR) [110]	3 nM (*n*, X) [73] 70 nM (*n*, H, *h*, FLIPR) [110]	No activity [73] >10,000 nM (*n*, H, *h*, FLIPR) [110]	888 nM (*n*, X) [73] >10,000 nM (*n*, H, *h*, FLIPR) [110]	323 nM (*n*, X) [73] >10,000 nM (*n*, H, *h*, FLIPR) [110]	>10,000 nM (*n*, H, *h*, FLIPR) [110]	5120 nM (*n*, H, *h*, FLIPR) [110]	55% block (*n*, X) [73] >10,000 nM (*n*, H, *h*, FLIPR) [110]		
CcoTx-2	1	407 nM (*n*, X) [73] 170 nM (*n*, H, *h*, FLIPR) [110]	8 nM (*n*, X) [73] 80 nM (*n*, H, *h*, FLIPR) [110]	88 nM (*n*, X) [73] 5570 nM (*n*, H, *h*, FLIPR) [110]	400 nM (*n*, X) [73] >10,000 nM (*n*, H, *h*, FLIPR) [110]	1634 nM (*n*, X) [73] >10,000 nM (*n*, H, *h*, FLIPR) [110]	3990 nM (*n*, H, *h*, FLIPR) [110]	230 nM (*n*, H, *h*, FLIPR) [110]	40% block (*n*, X) [73] >10,000 nM (*n*, H, *h*, FLIPR) [110]		
CcoTx-3	2	No activity [73]	No activity [73]	No activity [73]	No activity [73]	447 nM (*n*, X) [73]			45% block (*n*, X) [73]		
PaurTx3	1	610 nM (*n*, X) [73]	0.6 nM (*n*, X) [73]	42 nM (*n*, X) [73]	288 nM (*n*, X) [73]	72 nM (*n*, X) [73]			65% block (*n*, X) [73]		
Hm-1	9		32.4% block at 200 nM (*n*, X, *r*) [120]		336.4 nM (*n*, X, *r*) [120] 40% block at 200 nM (*n*, X, *r*) [120]	36.5% block at 200 nM (*n*, X, *h*) [120]	38.7% block at 200 nM (*n*, X, *m*) [120]				
Hm-2	9		64.6% block at 200 nM (*n*, X, *r*) [120]		154.8 nM (*n*, X, *r*) [120] 61.9% block at 200 nM (*n*, X, *r*) [120]	17.8% block at 200 nM (*n*, X, *h*) [120]	38.7% block at 200 nM (*n*, X, *m*) [120]				
GTx1-15	1			120 nM (*s*, H, *h*) [239]		No effect up to 2000 nM (*s*, H, *h*) [239]		7 nM (*s*, H, *h*) [239]	No effect up to 930 nM (*s*, H, *h*) [239]		
VSTx-3	1			190 nM (*s*, H, *h*) [239]		No effect up to 1000 nM (*s*, H, *h*) [239]		430 nM (*s*, H, *h*) [239]	770 nM (*s*, H, *h*) [239]		
Hd1a	1	87% block (*rec*, X, *h*) [121]	55% block (*rec*, X, *h*) [121]	23%–31% block (*rec*, X, *h*) [121]	23%–31% block (*rec*, X, *h*) [121]	No inhibition up to 1000 nM (*rec*, X, *h*) [121]	23%–31% block (*rec*, X, *h*) [121]	111 nM (*rec*, X, *h*) [121] 87% block (*rec*, X, *h*) [121]	No inhibition up to 1000 nM (*rec*, X, *h*) [121]		
Cd1a	1	2180 μM (*s*, H, *h*, FLIPR) [110]	130 μM (*s*, H, *h*, FLIPR) [110]	>30,000 nM (*s*, H, *h*, FLIPR) [110]	>30,000 nM (*s*, H, *h*, FLIPR) [110]	>30,000 nM (*s*, H, *h*, FLIPR) [110]	>30,000 nM (*s*, H, *h*, FLIPR) [110]	3340 nM (*s*, H, *h*, FLIPR) [110] 16 nM (*s*, H, *h*) [110]	6920 nM (*s*, H, *h*, FLIPR) [110]		
Pre1a	1	57.1 nM (*s*, H, *h*) [111]	189.6 nM (*s*, X, *r*) [111]	8000 nM (*s*, X, *r*) [111]	16.5% block at 1000 nM (*s*, X, *r*) [111]	8.6% block at 1000 nM (*s*, X, *r*) [111]	221.6 nM (*s*, H, *h*) [111]	114 nM (*s*, X, *r*) [111] 15 nM (*s*, H, *h*) [111]			
Pn3a	2	37 nM (*s*, H, *h*) [61]	124 nM (*s*, H, *h*) [61]	210 nM (*s*, H, *h*) [61] (	144 nM (*s*, H, *h*) [61]	800 nM (*s*, H, *h*) [61]	129 nM (*s*, H, *h*) [61])	0.9 nM (*s*, H, *h*) [61] 1.5 nM (*s*, H, *r*) [61] 4.4 nM (*s*, H, *m*) [61]	50,000 nM (*s*, H, *h*) [61]	2427 nM (*s*, H, *h*) [61]	
Hl1a	7	No inhibition (s, H_T_, *h)* [123]	No inhibition (s, H_T_, *h*) [123]	No inhibition (s, H_T_, *h*) [123]	No inhibition (s, H_T_, *h*) [123]	No inhibition (s, H_T_, *h*) [123]	No inhibition (s, H_T_, *h*) [123]	No inhibition (s, H_T_, *h*) [123]	2190 nM (*s*, ND7/23 cell, *h*) [123]		3760 nM (*s*, rat DRG) [123]
GpTx-1	1	6000 nM (*s*, H, *h*, FLIPR) [122]	5000 nM (*s*, H, *h*, FLIPR) [122]	20 nM (*s*, C, *h*) [76] 22,000 nM (*s*, H, *h*, FLIPR) [122]	301 nM (*s*, H, *h*, Manual ephys) [76] 200 nM (HEK293, *h*, synthetic, PatchXpress) [76] 326,000 nM (*s*, H, *h*, FLIPR) [122]	4200 nM (*s*, H, *h*, Manual ephys) [76] >10,000 nM (*s*, H, *h*, PatchXpress) [76] 140,000 nM (*s*, H, *h*, FLIPR) [122]	17,000 nM (*s*, H, *h*, FLIPR) [122]	4.4 nM (*s*, H, *h*, Manual ephys) [76] 10 nM (*s*, H, *h*, PatchXpress) [76] 580 nM (*s*, H, *h*, FLIPR) [122] 8 nM, open/inactivated state; 13 nM, closed/resting state (*s*, C, *h*,) [122]	12,200 nM (*s*, C, *h*, Manual ephys) [76] 68,000 nM (*s*, H, *h*, FLIPR) [122]		
F5A-M6F-T26L-K28R-GpTx-1	1				1900 nM (*s*, H, *h*, PatchXpress) [76]	>10,000 nM (*s*, H, *h*, PatchXpress) [76]		1.6 nM (*s*, H, *h*, PatchXpress) [76]			
Df1a	2	14.3 nM, *s-NH*_2_; 30.7 nM, *s-OH*; (H, *h*) [92]	1.9 nM, *s-NH*_2_; 3 nM, *s-OH*; (H, *h*) [92]	3 nM, *s-NH*_2_; 10 nM, *s-OH*; (H, *h*) [92]	24 nM, *s-NH*_2_; 53.6 nM, *s-OH*; (H, *h*) [92]	45.3 nM, *s-NH*_2_; 125.6 nM, *s-OH*; (H, *h*) [92]	7.6 nM, *s-NH*_2_; 23 nM, *s-OH*; (C, *h*) [92]	1.9 nM, *s-NH*_2_; 60.5 nM, *s-OH*; (H, *h*) [92]			
Phlo1a	2							459 nM (*n*, X, *h*) [240]			
Phlo1b	2							360 nM (*n*, X, *h*) [240]			
Phlo2a	3		404 nM (*n*, X, *h*) [240]			218 nM (*n*, X, *h*) [240]		333 nM (*n*, X, *h*) [240]			
GrTx-1	3	630 nM (*n*, H, *h*) [241]	230 nM (*n*, H, *h*) [241]	770 nM (*n*, H, *h*) [241]	1290 nM (*n*, H_T_, *h*) [241]	~22,000 nM (*n*, H, *h*) [241]	630 nM (*n*, H, *h*) [241]	370 nM (*n*, H_T_, *h*) [241]			
GsAFII	3	5700 nM (*n*, H, *h*) [241]	12,000 nM (*n*, H, *h*) [241]	24,000 nM (*n*, H, *h*) [241]	4000 nM (*n*, H_T_, *h*) [241]	~42,000 nM (*n*, H, *h*) [241]	6600 nM (*n*, H, *h*) [241]	1030 nM (*n*, H_T_, *h*) [241]			

Note: *s*:- synthetic; *n*:- native; *rec*:- recombinantly expressed; *rec-G*:- recombinantly expressed with an extra N-terminal Glycine; *s-OH*:- synthetic free carboxyl end; *s-NH*_2_:- synthetic C-terminal amidated; H:- HEK 293 cells; H_T_:- transiently transfected HEK 293 cells; X:- *Xenopus* oocytes; C:- CHO cells; S:- SHSY5Y cells; *h*:- human Na_V_; *r*:- rat Na_V_; *m*:- mouse Na_V_; *r*:- rat Na_V_; *M*:- mammalian Na_V_; FLIPR:- Fluorescence Imaging Plate Reader.

**Table A2:** Excitatory effects of spider knottins on Na_V_ channel subtypes. Values presented were obtained from electrophysiological experiments, unless otherwise as stated.

Toxin	NaSpTx Family	Na_V_1.1	Na_V_1.2	Na_V_1.3	Na_V_1.4	Na_V_1.5	Na_V_1.6	Na_V_1.7	Na_V_1.8	Na_V_1.9	Others
JzTx-I	7		870 nM (*n*, H_T_, *r*) [100]	845 nM (*n*, H_T_, *r*) [100]	339 nM (*n*, H_T_, *r*) [100]	335 nM (*n*, H_T_, *h*) [100]		348 nM (*n*, H_T_, *h*) [100]			130 nM (*n*, TTX-S currents, rat DRG); No inhibition in rat DRG TTX-R currents; 31 nM in rat cardiac myocytes) [101]
JzTx-II	7			1650 nM (*n*, H_T_, *r*) [98]		125 nM (*n*, H_T_, *h*) [98]					
Hm1a	2	38 nM (*s*, X, *h*) [20]	236 nM (*s*, X, *h*) [20]	220 nM (*s*, X, *h*) [20]	No inhibition up to 1000 nM (*s*, X, *h*) [20]	No inhibition up to 1000 nM (*s*, X, *h*) [20]	No inhibition up to 1000 nM (*s*, X, *h*) [20]	No inhibition up to 1000 nM (*s*, X, *h*) [20]	No inhibition up to 1000 nM (*s*, X, *h*) [20]		
PnTx2-1	6	122 nM (*n*, X, *r*) [147]	No inhibition up to 1000 nM (*n*, X, *r*) [147]	No inhibition up to 1000 nM (*n*, X, *r*) [147]	No inhibition up to 1000 nM (*n*, X, *r*) [147]	87 nM (*n*, X, *h*) [147]	No inhibition up to 1000 nM (*n*, X, *m*) [147]		101.1 nM (*n*, X, *r*) [147]		

Note: *s*:- synthetic; *n*:- native; H_T_:- transiently transfected HEK 293 cells; X:- *Xenopus* oocytes; *h*:- human Na_V_; *r*:- rat Na_V_; *r*:- rat Na_V_.
